# Paracentesis Thoracis

**Published:** 1874-04

**Authors:** Sydney Ringer


*THE PRACTITIONER—December,* 1873:

Paracentesis Thoracis.—*By Sydney Ringer, M.D.*—I am induced to publish these few cases because they assist to answer some important questions concerning pleurisy with effusion and the operation of paracentesis thoracis. They show, what indeed is well known, how slight a disturbance this operation causes, and what immense relief it affords. They show that the operation may be usefully employed in the febrile and non-febrile period of the disease, and that during fever the fluid may be withdrawn by the aspirator, and not accumulate again. They prove, moreover, that in some cases of febrile and non-febrile empyema it is sufficient to withdraw part of the fluid by the aspirator, and that the rest of the pus may disappear; and that it is not always necessay to lay open the chest in order that the pus may drain entirely away. Moreover, they show that in severe empyema the temperature may be normal, or scarcely at all raised, and in those cases accompanied by chronic fever the pus may be perfectly sweet.

